# Evaluating Auditory Localization Capabilities in Young Patients with Single-Side Deafness

**DOI:** 10.3390/audiolres15040085

**Published:** 2025-07-09

**Authors:** Alessandro Aruffo, Giovanni Nicoli, Marta Fantoni, Raffaella Marchi, Edoardo Carini, Eva Orzan

**Affiliations:** Institute for Maternal and Child Health—IRCCS Burlo Garofolo, 34137 Trieste, Italy; alessandro.aruffo@burlo.trieste.it (A.A.); marta.fantoni@burlo.trieste.it (M.F.); raffaella.marchi@burlo.trieste.it (R.M.); edoardo.carini@burlo.trieste.it (E.C.); eva.orzan@burlo.trieste.it (E.O.)

**Keywords:** single-sided deafness, unilateral hearing loss, spatial hearing, sound localization, head movement, children, virtual reality, auditory compensation, hemispheric asymmetry

## Abstract

Background/Objectives: Unilateral hearing loss (UHL), particularly single-sided deafness (SSD), disrupts spatial hearing in children, leading to academic and social challenges. This study aimed to (1) compare azimuthal sound-localization accuracy and compensatory strategies between children with single-sided deafness (SSD) and their normal-hearing (NH) peers within a virtual reality environment, and (2) investigate sound-localization performance across various azimuths by contrasting left-SSD (L-SSD) and right-SSD (R-SSD) groups. Methods: A cohort of 44 participants (20 NH, 24 SSD) performed sound localization tasks in a 3D virtual environment. Unsigned azimuth error (UAE), unsigned elevation error (UEE), and head movement distance were analyzed across six azimuthal angles (−75° to 75°) at 0°elevation. Non-parametric statistics (Mann–Whitney U tests, Holm–Bonferroni correction) compared performance between NH and SSD groups and within SSD subgroups (L-SSD vs. R-SSD). Results: The SSD group exhibited significantly higher UAE (mean: 22.4° vs. 3.69°, *p* < 0.0001), UEE (mean: 5.95° vs. 3.77°, *p* < 0.0001) and head movement distance (mean: 0.35° vs. 0.12°, *p* < 0.0001) compared with NH peers, indicating persistent localization deficits and compensatory effort. Within the SSD group, elevation performance was superior to azimuthal accuracy (mean UEE: 3.77° vs. mean UAE: 22.4°). Participants with R-SSD exhibited greater azimuthal errors at rightward angles (45°and 75°) and at −15°, as well as increased elevation errors at 75°. Hemifield-specific advantages were strongest at extreme lateral angles (75°). Conclusions: Children with SSD rely on insufficient compensatory head movements to resolve monaural spatial ambiguity in order to localize sounds. Localization deficits and the effort associated with localization task call for action in addressing these issues in dynamic environments such as the classroom. L-SSD subjects outperformed R-SSD peers, highlighting hemispheric specialization in spatial hearing and the need to study its neural basis to develop targeted rehabilitation and classroom support. The hemifield advantages described in this study call for further data collection and research on the topic.

## 1. Introduction

Unilateral hearing loss (UHL) affects approximately 1 to 3 per 1000 live births, with incidence increasing during childhood due to factors such as otitis media, head trauma, or viral infections [1,2]. Although historically perceived as less debilitating than bilateral hearing loss, UHL is now widely recognized as a condition with substantial functional and developmental consequences. Estimates suggest that up to 35% of children with UHL may experience academic difficulties and require additional educational support, despite having one normally functioning ear [1,3].

Among the various forms of UHL, the most severe is single-sided deafness (SSD), which is defined as a severe or profound hearing loss in one ear (pure-tone average, PTA > 70 dB HL) and normal hearing in the contralateral ear (PTA ≤ 25 dB HL) [4,5]. This definition underscores a pronounced auditory asymmetry, typically characterized by a loss exceeding 70 dB HL in one ear and a hearing threshold below 35 dB HL in the better ear. Therefore, children with SSD represent the subgroup of UHL patients with the highest degree of spatial hearing disruption.

Recent guidelines from the World Health Organization (WHO) have revised the classification of hearing loss severity to more accurately reflect functional impairment. According to the World Report on Hearing [6], severe hearing loss is now defined as a PTA between 65 and 79 dB HL—associated with the inability to perceive most conversational speech even when spoken loudly—while profound hearing loss is defined as a PTA between 80 and 94 dB HL, where only very loud sounds may be perceived and speech is typically unintelligible.

Children with UHL, and particularly those with SSD, frequently experience delays in speech and language development, reduced academic performance, and impaired sound localization [2,7]. The absence of binaural input hinders their ability to separate speech from background noise, which is especially problematic in classroom environments and social settings. Spatial hearing tasks such as identifying the origin of sounds are notably affected, limiting both communication efficacy and safety awareness in dynamic auditory environments [2,8].

These difficulties are magnified in educational settings, where children with UHL must rely on monaural hearing. This leads to disproportionate challenges in speech perception, academic engagement, and peer interaction [7,8]. Localization deficits increase cognitive load, especially in noisy or dynamic contexts, requiring children to exert more mental effort to follow auditory targets such as a moving teacher or to isolate meaningful sounds from background noise. Although strategies like seating the child with the better ear toward the teacher are commonly recommended, these do not fully compensate for the spatial hearing limitations or the increased listening fatigue associated with UHL [9].

Auditory localization is a critical component of real-world auditory function, enabling individuals to detect and respond to salient stimuli such as an approaching vehicle or a nearby speaker. This capacity relies on binaural integration of interaural time differences (ITDs) and interaural level differences (ILDs), the key mechanisms underlying horizontal sound localization [10,11,12]. In normal-hearing individuals, these cues provide a precise spatial representation of the auditory environment. Pinna-generated spectral-shape cues are essential for elevation localization, especially in resolving front–back ambiguities [13].

In cases of unilateral hearing loss, and particularly in SSD, the absence of input from one ear forces listeners to rely on monaural cues, such as the head shadow effect (HSE) and spectral filtering by the pinna [14,15]. The absence of spectral cues in single-sided deafness patients has been shown to severely impair their vertical localization performance, while allowing free head movements enhances the utilization of these cues and markedly improves elevation accuracy [16,17].

While some early studies proposed that monaural listeners could perform relatively well under ideal conditions, more recent findings have consistently shown persistent localization deficits [15,16,18]. Typically, these individuals show a systematic localization bias toward the hearing side, with improved performance only within the ipsilateral hemifield.

Furthermore, recent evidence suggests a right-ear advantage in spatial hearing tasks, with slightly better localization outcomes observed when the intact ear is on the right side. This effect is hypothesized to stem from hemispheric specialization in auditory spatial processing [19,20,21].

This study had two primary objectives. First, we compared spatial localization performance between children with SSD and NH peers, using accuracy rates and head movement distance as metrics to quantify both task success and compensatory effort. By testing SSD participants with hearing devices switched off, we isolated the natural compensatory role of the intact ear and evaluate whether localization accuracy improves on the hearing side relative to the impaired side. Second, we investigated lateralization effects by comparing left-sided SSD (L-SSD) and right-sided SSD (R-SSD) groups, hypothesizing that hemispheric dominance (e.g., right-hemisphere specialization for spatial processing) or directional biases (e.g., handedness) may modulate localization outcomes. These comparisons aim to clarify whether the side of impairment influences the efficacy of compensatory strategies and to identify potential neurocognitive mechanisms underlying asymmetries in spatial hearing.

## 2. Materials and Methods

The Institutional Review Board of the Institute for Maternal and Child Health IRCCS “Burlo Garofolo” (Trieste, Italy) approved this study under the “Ricerca Corrente 17/23” project. Informed consent was obtained from the parents of each participating individual. The study received approval from the local Ethics Committee (protocol number: 17/23) and was conducted in accordance with the 2013 Declaration of Helsinki.

Only children with IQ > 85 were included in the study.

We recruited a cohort of 20 normal-hearing participants aged 18–25 to validate the accuracy and reliability of our ambisonic testing environment [22,23].

A total of 44 participants were enrolled in this study, divided into two primary groups: the NH group (normal hearing) and the SSD group (single-sided deafness, hearing threshold >= 65 dB). The NH group consisted of 20 young adults with typical hearing (4 males, 16 females; mean age = 22.35 years, SD = 0.59). The SSD group (Table 1) included 24 children with SSD. Of these, 8 had left-ear deafness (3 males, 5 females; mean age = 9.75 years, SD = 3.45; mean hearing threshold = 95.13 dB HL, SD = 11.58), and 16 had right-ear deafness (5 males, 11 females; mean age = 11.75 years, SD = 3.57; mean threshold = 91.81 dB HL, SD = 13.93). With respect to the onset of hearing loss, 13 children had congenital SSD (5 males, 8 females; mean age = 10.08 years, SD = 3.59; mean hearing threshold = 89.46 dB HL, SD = 15.76). Four children had post-lingual onset (two males, two females; mean age = 11.75 years, SD = 3.86; mean hearing threshold = 94.00 dB HL, SD = 8.04), while seven had uncertain prelingual onset (one male, six females; mean age = 12.57 years, SD = 3.31; mean hearing threshold = 98.71 dB HL, SD = 7.54). Detailed information about the SSD group can be found in Table 1.

All participants confirmed right-handedness verbally and reported no motor and vestibular impairments. Prior to participation, all the participants were evaluated by a certified speech therapist to confirm their capability for performing the experimental tasks. Recruitment occurred from 23 May 2024 to 7 March 2025, and anonymized data became available to the authors on 10 March 2025.

### 2.1. Experimental Setup

**Figure 1:**
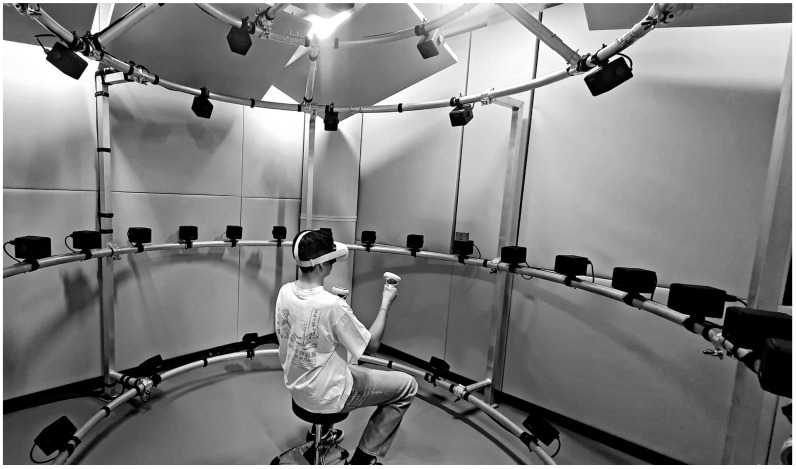
Experimental setup for multisensory spatial evaluation, Matilde Room, IRCCS Burlo Garofolo Trieste, Italy. Internal view of the Matilde Room during a test session. A participant is seated at the center of a semicircular array of loudspeakers wearing an Oculus Quest 2 head-mounted display and handheld controllers. The acoustically treated environment supports synchronized audio-visual stimulus presentation within a Unity3D-based virtual environment. (Parental informed consent was obtained for the participant portrayed in the image. The photograph is for exclusive use by IRCCS Burlo Garofolo and is not authorized for external distribution).

A three-dimensional (3D) virtual environment (VE) was implemented using Unity3D 5 software. Visual stimuli were presented through an Oculus Quest 2 head-mounted display (HMD), including Oculus Touch controllers (Meta Platforms Technologies Ireland Limited, Dublin, Ireland, Figure 1). The HMD tracked head positions in 3D space with submillimetre precision [24,25]. Orientation data, represented by quaternion values, were recorded with a ±1° precision at a sampling rate of 20 Hz. The data from the HMD were transmitted via the mqtt protocol over a 2.4 GHz Wi-Fi network.

A custom-developed application enabled real-time monitoring of head tracking and hand-pointing movements. Auditory stimuli were generated and controlled using a Max 8 (Cycling ‘74) software patch, output through a multi-channel amplifier connected via a MADIface USB 2.0 Audio Interface (RME GmbH, Heidelberg, Germany).

The acoustic reproduction setup comprised six loudspeakers arranged in a semicircular array with a radius of 1.5 m, positioned at angles of −75°, −45°, −15° in the left hemisphere, and 15°, 45°, and 75° in the right hemisphere relative to the initial stimulus position. The testing took place within a small enclosure measuring 3.4 × 3.4 m.

### 2.2. Room Acoustics and Reverberation Measurements

The experiment was conducted in a room acoustically treated to minimize reflections and modal resonances. Acoustic measurements identified distinct residual resonances with extended decay times between 180 Hz and 240 Hz, indicative of low-frequency modal behavior. Reverberation time (RT30) was measured using exponential sweep signals emitted sequentially from each of the six loudspeakers. Results demonstrated that RT30 values remained consistently below 250 ms for frequencies above 250 Hz, ensuring minimal reverberation. At 125 Hz, the reverberation time increased slightly, averaging approximately 365 ms, still within acceptable limits for spatial auditory tasks. Below 125 Hz, reverberation times notably increased, consistent with expected room mode limitations at lower frequencies.

Rooms with reverberation times (T60) below 0.3 s are typically categorized as acoustically dead environments, commonly seen in recording studios smaller than 50 m^3^ [26].

Participants were seated on a height-adjustable chair positioned at the focal point of the semicircle, with their head located 1.5 m from the loudspeakers at zero elevation.

Calibration procedures ensured alignment between virtual sound sources and corresponding physical loudspeakers, with a calibration precision of 1°, determined by the Oculus controller’s rotational accuracy [27].

### 2.3. Stimuli

The 100-ms linear onset was selected to reliably trigger the precedence effect, allowing participants to extract valid spatial cues even in a reverberant environment [28].

The acoustic stimuli consisted of pulsated pink noise characterized as follows:

- Attack: Linear amplitude ramp from 0% to 100% over 100 ms;
- Decay: 0 ms;
- Sustain: 100 ms at 100% amplitude;
- Release: Linear amplitude ramp from 100% to 0% over 100 ms.

The pink noise was selected due to its broadband characteristics, to enhance the accuracy of sound localization [29].

Stimuli were presented at 65 dB SPL (Z-weighted), measured at ear position using a calibrated sound-level meter (XL2 Sound Level Meter, NTi Audio).

Before each experimental session, seat height and interpupillary distance were adjusted to ensure accurate alignment of the loudspeakers with participant ear positions. Spatial alignment calibration was performed using visual markers in the virtual environment [30].

In our study, participants were instructed to respond rapidly and efficiently, although they were explicitly informed that, if additional time was necessary to achieve greater accuracy, they could take that extra time. This instructional approach aligns with previous findings [31,32], demonstrating that verbal instructions modulating the speed–accuracy trade-off influence decision-making by adjusting key cognitive parameters. Specifically, emphasizing speed reduces the effective decision threshold, increasing baseline cortical integrator neuron activity, thus producing faster yet less accurate responses. Conversely, accuracy-focused instructions raise decision thresholds, promoting slower but more precise responses. However, Ref. [33] reported that although instructions emphasizing speed led to marginally faster overall responses, they did not significantly affect the critical probe–irrelevant reaction time differences, suggesting that speed instructions do not undermine the essential mechanism underlying the response time concealed information test (RT-CIT).

The experimental protocol consisted of 48 trials. Thus, each participant was presented with 8 stimuli from each of the angles considered. Prior to data collection, participants completed a training phase consisting of five trials to familiarize themselves with the task.

## 3. Results

All statistical analyses were performed using Python version 3.12.8. Two custom scripts were developed to preprocess the data and perform inferential tests. The first script, written in Python, computed group-level descriptive statistics including mean, median, standard deviation, and interquartile range (IQR), and tested for normality using the Shapiro–Wilk test.

Given the non-normal distribution of the dependent variables (unsigned angular error and head distance), a second script implemented the Mann–Whitney U test to compare performance between groups. For each comparison, rank-biserial correlation (r_a_) was calculated to quantify effect size. All data parsing and computation routines were automated using Python’s standard libraries and scientific modules.

Holm–Bonferroni correction for multiple comparisons was applied within the scripts to adjust *p*-values and control for family-wise error rate. Data files were structured in annotated text format, and group-wise values were extracted and analyzed using custom parsing functions.

Participants were divided according to hearing status—normal hearing (NH) versus single-sided deafness (SSD)—and, within the SSD group, by the side of the hearing loss (right-SSD vs. left-SSD). The data were assessed both across specific angular conditions (−75°, −45°, −15°, 15°, 45°, 75°). Due to non-normal distributions confirmed via Shapiro–Wilk tests (*p* < 0.0001 in all subgroups), the Mann–Whitney U test was employed for all group comparisons.

### 3.1. Differences Between NH and SSD Groups

**Figure 2:**
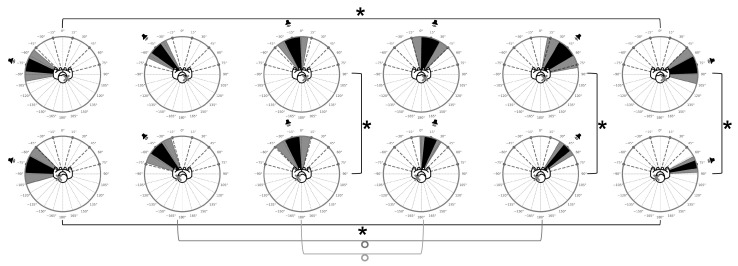
Each plot shows the position of the stimulus (speaker icon), the UAE represented by a black sector, and the corresponding standard deviation shown as a gray sector. The top row corresponds to the R-SSD group and the bottom row to the L-SSD group. The angles of stimulus presentation are arranged from left to right as follows: −75°, −45°, −15°, 15°, 45°, and 75°. Asterisks and bold brackets indicate within-group (L-SSD and R-SSD) and between-group (mirrored angle) comparisons that reached statistical significance, as detailed in the related analysis sections. Circular symbols and thin brackets denote comparisons that approached significance.

**Figure 3:**
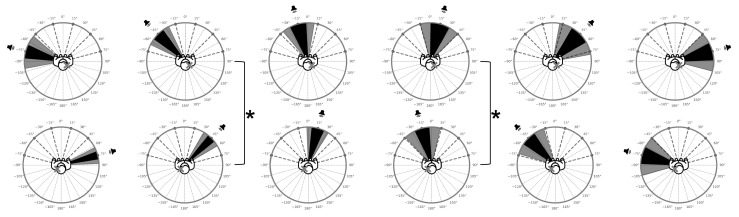
Between-group comparison of angular localization accuracy at mirrored stimulus positions for L-SSD and R-SSD participants. Each plot shows the position of the stimulus (speaker icon), the UAE represented by a black sector, and the corresponding standard deviation shown as a gray sector. The top row corresponds to the R-SSD group and the bottom row to the L-SSD group. This figure focuses on between-group comparisons at mirrored angles, aligning each stimulus location with the hearing side of the participants (e.g., −15° for L-SSD vs. 15° for R-SSD). Asterisks and bold brackets indicate statistically significant differences between the two groups as described in the corresponding analysis section.

**Figure 4:**
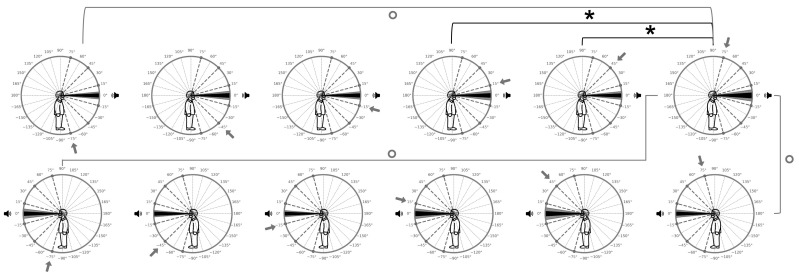
Between-group comparison of elevation localization accuracy for R-SSD (upper row) e and L-SSD (lower row) participants. Each plot displays the stimulus position in elevation (speaker icon) and its corresponding azimuthal angle (arrow pointing to the designated azimuth). Unsigned elevation error (UEE) is shown as a black sector, with its standard deviation in gray. The upper row represents the R-SSD group; the lower row represents the L-SSD group. Asterisks and bold brackets mark statistically significant differences between groups at the same angle, while circular symbols and thin brackets denote comparisons that approached significance.

The most robust and consistent effects emerged from comparisons between the NH and SSD groups, as shown in Table 2, Table 3 and Table 4. The analysis of the UAE values, UEE values, and head distance, without distinction by angle, reported statistically significant differences between the groups. For azimuthal error, the Mann–Whitney test revealed a highly significant effect (U = 205,723.5, *p* < 0.0001) with a large rank-biserial correlation (r_a_ = −0.6425). Similarly, elevation error differed significantly between groups (U = 529,896.0, *p* < 0.0001; r_a_ = −0.1979), with NH listeners again outperforming SSD participants. Finally, head-movement distance was also greater in the SSD group (U = 283,347.5, *p* < 0.0001; r_a_ = −0.5077). When broken down by angle, azimuthal error and head-movement comparisons were highly significant across all six angular conditions (*p* < 0.0001; see Figure 2 and Figure 3), whereas elevation error did not reach significance at 15° (*p* = 0.1151) or 45° (*p* = 0.0651; see Figure 4). Effect sizes for UAE ranged from −0.4364 to −0.8293, and for Head Distance from −0.2765 to −0.6733, indicating robust group-level divergence across all azimuths. In contrast, UEE effect sizes were smaller (−0.1034 to −0.2565), with minimal effects at 15° (−0.1034) and 45° (−0.1211). The consistent negative values suggest that SSD participants tended to display greater spatial dispersion or deviation compared with the NH group.

### 3.2. Differences Within Left-SSD and Right-SSD Groups

Accuracy scores at −75°, −45°, and −15° were compared with those at 15°, 45°, and 75° (Table 5). For the L-SSD group, a significant difference was observed between the hemifields (*p* = 0.0148) comparing 75° and −75° (Figure 2), with better performance on the hearing side. Marginally significant differences were obtained comparing −45°with 45° and −15° with 15°. Similarly, the R-SSD group also showed a significant difference (*p* = 0.0223) in favor of the hearing hemifield for the same angles range (Figure 2). No significant differences were observed when comparing combinations of other angles.

### 3.3. Differences Between Left-SSD and Right-SSD Groups

The most robust and consistent differences between the L-SSD and R-SSD groups emerged in the analysis of UAE and head distance, as summarized in Table 6 and Table 7 When all angles were considered together (All), UAE was significantly greater in the R-SSD group compared with the L-SSD group (*p* = 0.0004), with a positive rank-biserial correlation (r_a_ = 0.1234), indicating greater spatial deviation among R-SSD participants. Similarly, head distance (Table 7) was significantly higher in the R-SSD group (*p* = 0.0472; r_a_ = 0.0689), suggesting a greater extent of head movement during localization tasks.

When broken down by angle, specific conditions revealed stronger effects. For angular distance, the differences were significant at −15° (*p* = 0.0493, r_a_ = 0.1676), 45° (*p* = 0.0078, r_a_ = 0.2269), and 75° (*p* = 0.0001, r_a_ = 0.3257), consistently showing greater deviation in the R-SSD group on their impaired side. These results suggest angle-dependent spatial inaccuracy, particularly in the lateral and far-right fields for R-SSD participants.

Regarding head distance, significant differences were observed at −75° (*p* = 0.0009, r_a_ = −0.283), −45° (*p* = 0.0002, r_a_ = −0.3125), 15° (*p* = 0.0005, r_a_ = 0.2975), 45° (*p* < 0.0001, r_a_ = 0.4451), and 75° (*p* = 0.0001, r_a_ = 0.3401). The direction of the effect sizes indicates that R-SSD participants exhibited greater head movement for rightward angles (positive r_a_), while L-SSD participants showed increased movement for leftward angles (negative r_a_), reflecting compensatory behaviors relative to the impaired side.

Further analysis was set to evaluate differences in accuracy between the two groups (L-SSD and R-SSD) at corresponding asymmetrical angles, based on the side of hearing impairment. To isolate the impact of the hearing-impaired side, between-group comparisons were performed at asymmetrical angles that mirrored the spatial position relative to the intact ear (Figure 3). For example, localization accuracy at −15° in L-SSD participants (corresponding to the non-impaired side) was compared with 15° in R-SSD participants (non-impaired side), and similarly for all the angles’ combinations. This approach allowed for direct assessment of group-specific performance when stimuli were positioned on the hearing side.

Two comparisons yielded statistically significant differences. The contrast between −15° (L-SSD) and 15° (R-SSD) showed a significant effect (U = 3591.5, *p* = 0.0097, r_a_ = −0.2206), with lower angular distance in the L-SSD group. Similarly, the comparison between 45° (L-SSD) and −45° (R-SSD) also reached significance (U = 3697.5, *p* = 0.0205, r_a_ = −0.1976). No other asymmetrical angle comparisons showed significant effects, although the comparison between 75° (L-SSD) and −75° (R-SSD) approached significance (U = 3867.0, *p* = 0.0591, r_a_ = −0.1608), suggesting a possible trend that warrants further investigation.

### 3.4. Elevation Localization: Within- and Between-Group Comparisons at Individual Angles for L-SSD and R-SSD

- As shown in Table 8, no individual angle comparisons between R-SSD and L-SSD reached statistical significance. To probe angle-specific effects in elevation across both SSD groups, we conducted pairwise Mann–Whitney U tests on the unsigned elevation error (UEE) value for every tested angle within each group and between groups. At +75° in R-SSD participants (mean UEE = 7.7734°, SD = 9.1585°), we observed statistically significant elevation errors, compared with +15° R-SSD (U = 6939.0, *p* = 0.0337, r_a_ = 0.1530) and +45° R-SSD (U = 7029.5, *p* = 0.0489, r_a_ = 0.1419).

Between-group comparisons also revealed trends toward significance at +75° R-SSD versus L-SSD, including results as follows:

- +75° L-SSD (U = 4801.5, *p* = 0.0512, r_a_ = 0.1722)
−75° L-SSD (U = 4742.5, *p* = 0.0740, r_a_ = 0.1578)
−15° L-SSD (U = 7147.0, *p* = 0.0767, r_a_ = 0.1276)

All positive rank-biserial correlations indicated that UEE at +75° in R-SSD exceeded that at all other angles, underscoring a focal elevation deficit at the most ipsilateral position (see Figure 4).

## 4. Discussion

The results of this study demonstrate that participants with SSD exhibited significantly lower accuracy rates for sound source azimuthal localization compared with participants with no hearing impairment (NH). This disparity was observed considering both the general performance and in the analysis of the accuracy rates for each angle considered in the analysis. These findings align with existing literature, reinforcing those children with SSD face persistent challenges in spatial hearing tasks [15,18,34]. When comparing azimuthal and elevation localization between normal-hearing and SSD participants using stimuli fixed at 0° elevation, we found significant localization deficits in the SSD group along both dimensions [35]. However, the elevation errors were markedly smaller than the azimuthal errors for SSD listeners, underscoring the greater reliance of vertical localization on monaural spectral cues at 0° elevation. Angle-by-angle analyses revealed that SSD–NH differences were significant at −75°, −45°, −15°, and 75° in elevation, while mid-range azimuths (15° and 45°) showed no significant group differences. This suggests that decomposing performance by individual angles offers a more nuanced understanding of localization abilities in SSD patients. Unlike our earlier analyses comparing UAE and head-movement metrics between groups, we performed a detailed, angle-by-angle examination of elevation errors across all tested positions. This exploratory approach revealed that only the R-SSD group exhibited significant elevation deficits for sounds originating in the right hemifield—even though all stimuli were presented at 0° elevation. These findings underscore the value of within- and between-group, angle-specific analyses for uncovering localized impairments that broader comparisons may overlook.

All of our participants underwent a comprehensive neurological examination to rule out motor impairments. In addition, participants older than five years received both clinical vestibular testing and video head impulse testing (vHIT) to exclude vestibular dysfunction as a confounding factor, since vestibular deficits can lead to less efficient movement strategies, characterized by longer response times and increased head rotations, that can compromise localization precision [36].

Notably, alongside these challenges in the accuracy rates, the SSD group showed increased head movement distances. Children with SSD consistently generated larger head movements during localization efforts, suggesting a compensatory reliance on the head shadow effect to enhance interaural level differences (ILDs) and spectral cues. Our results corroborate prior evidence that this adaptive strategy remains suboptimal, as exclusive dependence on head shadowing does not fully resolve localization deficits [15]. The observed increase in head motion underscores how orienting in an auditory space is more demanding for children with SSD. These results align with evidence that unilateral hearing loss disrupts binaural cues critical for azimuthal localization, forcing reliance on monaural spectral cues and compensatory head movements to resolve ambiguity [15]. As such the results presented here emphasize and reinforce the clinical need to develop targeted interventions (e.g., auditory training programs or assistive technologies) that address localization challenges, particularly in dynamic environments such as classrooms, where sound sources (e.g., a moving teacher) demand continuous spatial recalibration [8,9].

Normal-hearing individuals exhibit significant ipsilateral–contralateral differences in source-waveform latency and amplitude [37]; contralateral activity is consistently larger and peaks earlier than ipsilateral activity regardless of the stimulated ear. In contrast, unilaterally deaf subjects showed a selective reduction in interhemispheric amplitude differences that depended on the side of deafness, while latencies remained symmetric across hemispheres. Notably, these amplitude alterations were confined to participants with profound left-ear deafness. This lateralized amplitude effect could be related to our findings, where we observed hemifield-specific localization advantages. Within-group comparisons revealed a lateralized advantage for the intact hemifield in both SSD groups. For L-SSD participants, this advantage was broadly observed across the hearing (right) side but reached statistical significance only at extreme lateral angles (−75° vs. 75°), with mid-range angles (±45°, ±15°) showing marginally significant differences. In contrast, R-SSD participants exhibited a significant lateralized advantage exclusively at extreme angles (75° vs. −75°), with no detectable benefit at central positions. These findings align with prior evidence that monaural hearing support localization accuracy ipsilateral to the intact ear [14,15]. The pronounced advantage at 75° could be explained by reduced spectral ambiguity at lateral angles, where head shadow effects and pinna-derived cues are more salient [14]. To optimize auditory access in classrooms, children with SSD should be positioned with their intact ear oriented toward primary speech sources (e.g., teachers). This configuration leverages natural spectral cues while mitigating compensatory head movements, thereby reducing cognitive load during dynamic listening tasks. The weaker lateralized advantage in the R-SSD group may have stemmed from right-hemisphere dominance in spatial processing, which could amplify contralateral (rightward) deficits despite compensatory strategies. This hypothesis is further explored in the following paragraphs.

### Lateralized Spatial Processing Asymmetries in SSD

The findings revealed angle-dependent differences between L-SSD and R-SSD participants in both angular error and head movement measures. Specifically, the L-SSD group exhibited significantly lower azimuthal error than the R-SSD group at rightward angles (45° and 75°), reflecting a spatial processing advantage on their hearing side. Interestingly, this lateralized advantage was not mirrored in the R-SSD group, who did not demonstrate improved localization on the leftward side, as might have been expected. Moreover, at the left-central angle, L-SSD participants outperformed their R-SSD counterparts, suggesting further asymmetry in spatial performance.

A complementary analysis comparing localization accuracy at asymmetrical angles—defined here as positions equidistant from the midline but opposite in side relative to the deaf ear (e.g., −75° and 75°)—revealed that R-SSD participants performed more poorly on their hearing side (right) than L-SSD participants did on theirs (left), particularly at 45° and, to a lesser extent, 75°. In addition, the L-SSD group outperformed the R-SSD group in localizing sounds at −15° from their hearing side (i.e., 15° on the impaired side).

Taken together, these findings suggest lateralized asymmetry in spatial performance associated with the side of single-sided deafness. This pattern may reflect underlying hemispheric specialization, with the right hemisphere—known for its dominance in spatial processing—providing a relative advantage to L-SSD individuals while amplifying deficits in R-SSD cases. This interpretation aligns with previous research [20,21]. Future studies should further investigate whether this asymmetry extends to broader aspects of functioning in individuals with SSD.

## 5. Conclusions and Limits

This study highlights the marked differences in sound localization performance between children with SSD and their peers with normal hearing, emphasizing the increased cognitive effort required for spatial hearing in SSD. The significantly greater head movement distances observed in the SSD group suggest that children with unilateral hearing loss rely heavily on compensatory strategies—such as enhancing ILDs through head motion—to overcome limitations in binaural auditory cues. While adaptive, these strategies place additional cognitive demands on the listener, particularly in dynamic environments like classrooms. Therefore, assessing localization abilities should become an integral component of audiological evaluations, especially in educational contexts, to inform interventions aimed at reducing cognitive load and improving learning outcomes.

Additionally, the observed lateralized asymmetries—most notably the superior localization performance in L-SSD compared with R-SSD—underscore the need for further investigation into the role of hemispheric specialization in spatial auditory processing. Understanding the neural mechanisms underlying these asymmetries could guide the development of tailored rehabilitation strategies and educational accommodations for children with SSD.

These conclusions should be considered in light of some limitations. First, there was an age mismatch between the groups. The SSD participants were children (mean age = 12.03 years), whereas the NH control group comprised young adults (mean age = 22.35 years). Although previous research [22,38] suggests that spatial hearing matures by early adolescence, developmental differences in auditory experience or executive functioning cannot be entirely ruled out. This NH cohort was selected based on prior evidence that localization performance peaks in adults aged 18–25 [22,23], thereby providing an optimal benchmark for comparing the spatial-localization abilities of our SSD participants using the same apparatus. Notably, the NH group’s performance aligns well with existing pediatric norms, supporting the relevance of the comparison. Second, the study did not evaluate SSD participants using hearing-assistive devices, which could potentially improve localization accuracy and reduce compensatory head movements.

Third, in the present study, we analyzed participants by dividing them into two main groups: NH (normal hearing) and SSD (single-sided deafness). While this grouping aligns with the study’s primary aim, recent findings by Federici et al. [39] suggest that the timing of onset (i.e., congenital vs. acquired forms) may have important implications for auditory development and cortical plasticity, particularly in cases of bilateral hearing loss. However, since our study focused specifically on unilateral hearing loss, we chose to consider the SSD population as a single group, regardless of onset type. To minimize the test duration, we restricted stimuli to a single elevation plane (0°) and maximized azimuthal spacing between loudspeakers. As a result, our paradigm did not probe elevation cues at varying heights. Future studies should undertake a dedicated investigation of elevation localization in SSD participants, systematically sampling across both azimuthal and elevational angles and conducting angle-by-angle comparisons to elucidate compensatory strategies and inform advanced fitting or rehabilitation protocols. Moreover, future research with a larger sample will be necessary to investigate whether the timing of SSD onset affects spatial-hearing outcomes and to evaluate the potential benefits of further subgrouping within SSD populations. Concurrently, studies should examine how these individual factors interact with real-world auditory demands such as those encountered in classrooms, by assessing both spatial hearing and the cognitive load associated with monaural listening under ecologically valid conditions.

## Figures and Tables

**Table 1:** Clinical characteristics of the patient cohort.

Patient ^1^	Age	Sex	Side ^2^	Threshold ^3^	Onset ^4^	Type ^5^	Etiology ^6^
1	7	Male	Left	100	Congenital	SHL	CN VIII hyp.
2	17	Male	Left	110	Congenital	SHL	IP-II
3	6	Female	Left	72	Congenital	MHL	A.A.
4	8	Female	Left	86	Congenital	SHL	CN VIII hyp.
5	9	Female	Left	100	Congenital	SHL	IP-I
6	10	Male	Left	93	Post-lingual	SHL	CN VIII hyp.
7	9	Female	Left	100	Uncertain Prelingual	SHL	Unknown
8	12	Female	Left	100	Post-lingual	SHL	SSNHL
9	8	Female	Right	65	Congenital	CHL	A.A.
10	9	Male	Right	77	Congenital	MHL	OAV syndrom
11	12	Male	Right	108	Congenital	SHL	CN VIII hyp.
12	10	Female	Right	100	Congenital	SHL	CMV
13	15	Female	Right	100	Congenital	SHL	CN VIII hyp.
14	14	Male	Right	75	Congenital	MHL	A.A.
15	11	Female	Right	100	Congenital	SHL	Unknown
16	11	Female	Right	108	Uncertain Prelingual	SHL	Unknown
17	14	Female	Right	83	Uncertain Prelingual	SHL	Tymp. plast.
18	12	Female	Right	100	Uncertain Prelingual	SHL	Unknown
19	15	Male	Right	100	Uncertain Prelingual	SHL	Unknown
20	17	Female	Right	100	Post-lingual	SHL	Tymp. plast.
21	18	Female	Right	100	Uncertain Prelingual	SHL	Unknown
22	8	Male	Right	83	Post-lingual	SHL	SSNHL
23	9	Female	Right	100	Uncertain Prelingual	SHL	SSNHL
24	5	Female	Right	70	Congenital	MHL	Microtia

^1^ SSD patient number, ^2^ side of the impaired ear (Left/Right), ^3^ Hearing threshold (pure Tone Average calculated across 500 Hz, 1000 Hz, 2000 Hz, and 4000 Hz frequencies expressed in decibels hearing level, ^4^ onset of hearing loss (categorized as congenital, uncertain prelingual, or post lingual), ^5^ type of hearing loss classified as sensorineural hearing loss (SHL), conductive hearing loss (CHL), or mixed hearing loss (MHL), based on audiological assessment, etiology ^6^: Auricular atresia (A.A.), microtia, suspected oculo-auriculo-vertebral spectrum (OAVS), sudden sensorineural hearing loss (SSNHL), tympanoplasty for cholesteatoma (Tymp. plast.), cytomegalovirus (CMV), unknown, hypoplasia of the eighth cranial (vestibulocochlear) nerve (CN VIII hyp.), incomplete partition type I (IP-I), incomplete partition type II (IP-I).

**Table 2:** Summary of mean unsigned azimuthal error (UAE) values and statistical significance for NH subjects and SSD patients across tested angular positions.

Angle ^1^	NH ^2^	SSD ^3^	*p*-Value ^4^	r_a_ ^5^
ALL	Mean: 3.69	Mean: 22.4	<0.0001	−0.6425
	SD: 3.6	SD: 29.7		
−75°	Mean: 5.1	Mean: 22.13	<0.0001	−0.4364
	SD: 4.04	SD: 33.02		
−45°	Mean: 3.97	Mean: 20.31	<0.0001	−0.6176
	SD: 3.06	SD: 25.02		
−15°	Mean: 3.2	Mean: 23.50	<0.0001	−0.7091
	SD: 2.68	SD: 28.76		
15°	Mean: 2.94	Mean: 24.58	<0.0001	−0.8293
	SD: 2.18	SD: 27.71		
45°	Mean: 2.99	Mean: 23.18	<0.0001	−0.6696
	SD: 4.04	SD: 34.22		
75°	Mean: 3.93	Mean: 20.78	<0.0001	−0.5697
	SD: 4.56	SD: 28.53		

^1^ Stimulus presentation azimuthal angle expressed in degrees (from −75° to +75°), including an aggregated “ALL” condition representing the average across all angles, ^2^ mean and standard deviation of azimuthal-localization performance for the normal-hearing group, ^3^ mean and standard deviation of the same measure in patients with SSD, ^4^ significance level of the statistical comparison between NH and SSD groups at each angle, ^5^ correlation coefficient.

**Table 3:** Summary of mean head movement distance (in degrees) and corresponding statistical comparisons between normal-hearing (NH) subjects and patients with single-sided deafness (SSD) across stimulus presentation angles.

Angle ^1^	NH ^2^	SSD ^3^	*p*-Value ^4^	r_a_ ^5^
ALL	Mean: 0.12	Mean: 0.35	<0.0001	−0.5077
	SD: 0.07	SD: 0.38		
−75°	Mean: 0.17	Mean: 0.35	<0.0001	−0.2765
	SD: 0.06	SD: 0.39		
−45°	Mean: 0.12	Mean: 0.32	<0.0001	−0.4497
	SD: 0.05	SD: 0.34		
−15°	Mean: 0.07	Mean: 0.38	<0.0001	−0.6733
	SD: 0.05	SD: 0.42		
15°	Mean: 0.07	Mean: 0.32	<0.0001	−0.6505
	SD: 0.05	SD: 0.38		
45°	Mean: 0.12	Mean: 0.31	<0.0001	−0.5077
	SD: 0.06	SD: 0.31		
75°	Mean: 0.16	Mean: 0.4	<0.0001	−0.4415
	SD: 0.07	SD: 0.39		

^1^ Stimulus presentation angle, expressed in degrees (from −75° to +75°), including an aggregated “ALL” condition representing the average across all angles. For each angle, the table reports the mean and standard deviation of head movement distance for the normal-hearing (NH) ^2^ group and for patients with single-sided deafness (SSD) ^3^. It also includes the significance level of the statistical comparison between NH and SSD groups at each angle ^4^, as well as the correlation coefficient ^5^.

**Table 4:** Summary of mean unsigned elevation error (UEE) values and statistical significance for NH subjects and SSD patients across tested azimuthal positions.

Angle ^1^	NH ^2^	SSD ^3^	*p*-Value ^4^	r_a_ ^5^
ALL	Mean: 3.77	Mean: 5.95	<0.0001	−0.1979
	SD: 4.41	SD: 7.26		
−75°	Mean: 3.10	Mean: 5.50	0.0006	−0.2257
	SD: 2.87	SD: 6.52		
−45°	Mean: 3.58	Mean: 5.84	0.0001	−0.2565
	SD: 4.07	SD: 6.08		
−15°	Mean: 3.09	Mean: 5.28	0.0001	−0.2525
	SD: 3.67	SD: 5.46		
15°	Mean: 4.43	Mean: 6.25	0.1151	−0.1034
	SD: 4.85	SD: 8.26		
45°	Mean: 4.52	Mean: 5.89	0.0651	−0.1211
	SD: 5.56	SD: 8.31		
75°	Mean: 3.93	Mean: 6.94	0.0003	−0.2347
	SD: 4.75	SD: 8.34		

^1^ Stimulus presentation azimuthal angle expressed in degrees (from −75° to +75°), including an aggregated “ALL” condition representing the average across all azimuthal angles, ^2^ mean and standard deviation of elevation-localization performance for the normal-hearing group, ^3^ mean and standard deviation of the same measure in patients with SSD, ^4^ significance level of the statistical comparison between NH and SSD groups at each angle, ^5^ correlation coefficient.

**Table 5:** Within-group comparisons of UAE between impaired and non-impaired hemifields for L-SSD and R-SSD participants.

Group	UAE Angle Comparison ^1^	Mean ± SD (°) ^2^	*p*-Value	r_a_
L-SSD	−75° vs. 75°	25.69 ± 36.11 vs. 11.43 ± 13.88	0.0148	0.2353
	−45° vs. 45°	22.68 ± 32.42 vs. 12.46 ± 16.47	0.0822	0.1678
	−15° vs. 15	22.15 ± 34.70 vs. 18.61 ± 15.63	0.0664	−0.1773
R-SSD	−75° vs. 75°	20.13 ± 31.13 vs. 26.08 ± 33.05	0.0223	−0.1655
	−45° vs. 45°	18.97 ± 19.71 vs. 29.22 ± 39.77	0.3729	−0.0645
	−15° vs. 15	24.26 ± 24.92 vs. 27.95 ± 32.18	0.3975	−0.0612

^1^ The table reports UAE (± standard deviation) for each mirrored angle pair (e.g., −75° vs. 75°), ^2^ mean and standard deviation, *p*-values and rank-biserial correlations (r_a_) are provided for each comparison.

**Table 6:** Comparison of UAE between L-SSD and R-SSD groups across spatial angles.

Angle ^1^	L-SSD ^2^	R-SSD ^3^	*p*-Value ^4^	r_a_^5^
ALL	Mean: 18.80	Mean: 24.40	0.0004	0.1234
	SD: 27.00	SD: 30.90		
−75°	Mean: 25.69	Mean: 20.13	0.3578	−0.0785
	SD: 36.11	SD: 31.13		
−45°	Mean: 22.68	Mean: 18.97	0.8475	0.0165
	SD: 32.42	SD: 19.71		
−15°	Mean: 22.15	Mean: 24.25	0.0493	0.1676
	SD: 34.70	SD: 24.92		
15°	Mean: 18.61	Mean: 27.95	0.2556	0.0970
	SD: 15.63	SD: 32.18		
45°	Mean: 12.45	Mean: 29.22	0.0078	0.2269
	SD: 16.46	SD: 39.77		
75°	Mean: 11.43	Mean: 26.08	0.0001	0.3257
	SD: 13.88	SD: 33.05		

^1^ Stimulus presentation angle, expressed in degrees (from −75° to +75°), including an aggregated “ALL” condition representing the average across all angles, mean and standard deviation, ^4^ significance level of the statistical comparison between L-SSD ^2^ and R-SSD ^3^ groups at each angle, correlation coefficient ^5^.

**Table 7:** Comparison of head distance between L-SSD and R-SSD groups across spatial angles.

Angle ^1^	L-SSD ^2^	R-SSD ^3^	*p*-Value ^4^	r_a_^5^
ALL	Mean: 0.31	Mean: 0.37	0.0472	0.0689
	SD: 0.33	SD: 0.39		
−75°	Mean: 0.41	Mean: 0.31	0.0009	−0.2830
	SD: 0.39	SD: 0.39		
−45°	Mean: 0.33	Mean: 0.31	0.0002	−0.3125
	SD: 0.25	SD: 0.38		
15°	Mean: 0.25	Mean: 0.36	0.0005	0.2975
	SD: 0.32	SD: 0.41		
45°	Mean: 0.21	Mean: 0.37	<0.0001	0.4451
	SD: 0.24	SD: 0.33		
75°	Mean: 0.29	Mean: 0.46	0.0001	0.3401
	SD: 0.31	SD: 0.41		

^1^ Stimulus presentation angle, expressed in degrees (from −75° to +75°), including an aggregated “ALL” condition representing the average across all angles, mean and standard deviation, significance level of the statistical ^4^ comparison between L-SSD ^2^ and R-SSD ^3^ groups at each angle, correlation coefficient ^5^.

**Table 8:** Comparison of UEE between L-SSD and R-SSD groups across spatial angles.

Angle ^1^	L-SSD ^2^	R-SSD ^3^	*p*-Value ^4^	r_a_^5^
ALL	Mean: 5.82	Mean: 6.01	0.8526	−0.0067
	SD: 7.46	SD: 7.16		
−75°	Mean: 5.79	Mean: 5.35	0.3361	−0.0850
	SD: 8.47	SD: 5.31		
−45°	Mean: 5.34	Mean: 6.09	0.7202	0.0317
	SD: 4.42	SD: 6.76		
−15°	Mean: 4.84	Mean: 5.50	0.8899	−0.0123
	SD: 4.52	SD: 5.89		
15°	Mean: 6.79	Mean: 5.98	0.1511	0.1267
	SD: 8.06	SD: 8.38		
45°	Mean: 6.90	Mean: 5.38	0.5489	0.0530
	SD: 11.00	SD: 6.56		
75°	Mean: 5.26	Mean: 7.77	0.0512	−0.1722
	SD: 6.11	SD: 9.15		

^1^ Stimulus presentation angle, expressed in degrees (from −75° to +75°), including an aggregated “ALL” condition representing the average across all angles, mean and standard deviation, ^4^ significance level of the statistical comparison between L-SSD ^2^ and R-SSD ^3^ groups at each angle, correlation coefficient ^5^.

## Data Availability

The data supporting the findings of this study are not publicly available due to privacy and ethical restrictions.

## Abbreviations

The following abbreviations are used in this manuscript:

CHL	Conductive hearing loss
HMD	Head-mounted display
HSE	Head shadow effect
ILD	Interaural level difference
ITD	Interaural time difference
R-SSD	Right-sided single-sided deafness
L-SSD	Left-sided single-sided deafness
MHL	Mixed hearing loss
MQTT	Message-queuing telemetry transport
NH	Normal hearing
PTA	Pure tone average
RT30	Reverberation time over 30 dB decay
SD	Standard deviation
SHL	Sensorineural hearing loss
SSD	Single-sided deafness
T60	Reverberation time over 60 dB decay
UAE	Unsigned azimuth error
UEE	Unsigned elevation error
UHL	Unilateral hearing loss
VE	Virtual environment
